# Scorpion Peptides and Ion Channels: An Insightful Review of Mechanisms and Drug Development

**DOI:** 10.3390/toxins15040238

**Published:** 2023-03-24

**Authors:** Lais Campelo Mendes, Gabriela Magnólia Melo Viana, Ana Leonor Abrahão Nencioni, Daniel Carvalho Pimenta, Emidio Beraldo-Neto

**Affiliations:** 1 Programa de Pós-Graduação em Ciências—Toxinologia do Instituto Butantan, São Paulo 05503-900, Brazil; 2 Laboratório de Bioquímica do Instituto Butantan, São Paulo 05503-900, Brazil; 3 Laboratório de Farmacologia do Instituto Butantan, São Paulo 05503-900, Brazil

**Keywords:** neurotoxins, scorpion toxins, sodium channel, potassium channel, calcium channel, chloride channel, transient receptor potential

## Abstract

The Buthidae family of scorpions consists of arthropods with significant medical relevance, as their venom contains a diverse range of biomolecules, including neurotoxins that selectively target ion channels in cell membranes. These ion channels play a crucial role in regulating physiological processes, and any disturbance in their activity can result in channelopathies, which can lead to various diseases such as autoimmune, cardiovascular, immunological, neurological, and neoplastic conditions. Given the importance of ion channels, scorpion peptides represent a valuable resource for developing drugs with targeted specificity for these channels. This review provides a comprehensive overview of the structure and classification of ion channels, the action of scorpion toxins on these channels, and potential avenues for future research. Overall, this review highlights the significance of scorpion venom as a promising source for discovering novel drugs with therapeutic potential for treating channelopathies.

## 1. Introduction

Venoms of arthropods are arsenals of bioactive molecules with applications in diverse resources [1]. Considering the arthropods of medical interest, scorpions of the family Buthidae are a rich source of useful peptides. They can be divided into two geographic groups. Old World scorpions are found in areas of northern Africa, southern Europe, Asia, and the Middle East and are represented by the genera *Androctonus*, *Leiurus*, *Mesobuthus*, *Hemiscorpius*, and *Buthus*; New World scorpions are found in the Americas and belong to the genera *Centruroids* and *Tityus* [2,3,4]. These two groups differ in their venom composition, the clinical symptomatology and severity of their envenomation, as well as the therapeutic approaches to their symptoms [5].

The scorpion venom is composed mostly of peptides, lipids, and low molecular-weight components [6]. Peptides act by modulating ion channels, which are responsible for performing essential functions in the body, such as neuronal signaling, muscle contraction, and hormone secretion, and may be targeted for the development of new drugs [6,7].

Toxins present in scorpion venom share physicochemical properties and homologous structures, and despite this homology, they have different pharmacological effects, which have developed over millions of years through evolution and the action of natural selection, favoring animals with neurotoxins that can block or modulate ion channels [8,9]. The interaction between ion channels and neurotoxins is the main *modus operandi* responsible for their pharmacological effects [8].

In this aspect, natural selection has led to the occurrence of mutations present in these channels, which may be responsible for the loss or gain of function and lead to the conduction of fewer or more ions, respectively; this aspect is fundamental for their activity [10]. Peptides acting on ion channels are found in the common ancestor of scorpions, suggesting that their fundamental and functional role in envenomation during evolution, especially when it comes to venoms of the family Buthidae, whose neurotoxins are highly toxic and display variability in their affinity towards ion channels found in mammals and arthropods, as well as within and between species [11].

Allied with the development of new technologies, help in the search for selective ligands using techniques such as transcriptomics, genomics, and mutagenesis that demonstrate changes suffered over the years and the possibility of changing its selectivity and affinity for a specific receptor [10,12]. The work recently carried out by the group of Santibáñez-López revealed important data on the relationship between the diversity of toxins and action on sodium channels, revealing that the origins of toxins directed to mammals are related to the basal line scorpions, being predators of mammals such as shrews, bats, and rodents, still demonstrate an evolutionary model for the diversification of sodium channel toxins in relation to their predators. The group further highlights the gaps that permeate this area, such as the difficulty in finding robust transcriptomes for this type of analysis [12]. An interesting aspect to be addressed when dealing with cases of envenomation is that scorpion stings cause intense local pain in the victims, who may eventually develop systemic poisoning, leading to cardiovascular and respiratory consequences due to the action of neurotoxins on the ion channel of the peripheral nervous system, resulting in a massive release of sympathetic and parasympathetic neurotransmitters [4,13].

A dysfunction in sodium, potassium, chloride, calcium, or transient receptor potential channels can lead to various neurological disturbances, including pain and depression, cardiovascular alterations, and cancer [14]. Ion channels also contribute to the different phases of the action potential and are involved in metabolic diseases, such as disorders of glucose homeostasis (hyperinsulinemia, hypoglycemia, and different forms of diabetes mellitus), becoming a challenge for studies and for the development of new drugs [15,16,17,18,19]. Dysfunctions of ion channels are called channelopathies and can be caused by deregulation in the opening and closing of the channel, a decrease in the amplitude of the currents, or the proteins that control its function. They arise from genetic mutations, the activity of periods of excitability in the cells, and the overexpression of channels that can induce tumor formation [8,10,20]. The nervous system depends on the functioning of pore structures of ion channels, which modulate the traffic of ions through the cell membrane and the regulation of the firing and propagation of action potentials. In this way, neurological diseases can arise from a deregulated expression or function of pore components [21].

Neurotoxins with action on ion channels are a target of interest for new drug discovery aiming for channelopathy treatment [22].

Given the relevance of ion channels and their importance in physiological and biological processes, studies on the characterization of their structures as well as their ligands have shown to be extremely important, since some compounds can be used in bioprospecting for new drugs. The main scorpion peptides that will be addressed during this review are presented in Table 1, as described below. However, there is still a long way to go for these peptides or their synthetic analogs to reach clinical phase studies. With that, we will approach in this review an overview of ion channels and their main scorpion peptides of interest, presenting their structures and their physiological effects, as well as their contribution to the future of drug development studies.

## 2. Structure of Ion Channels

**Figure 1:**
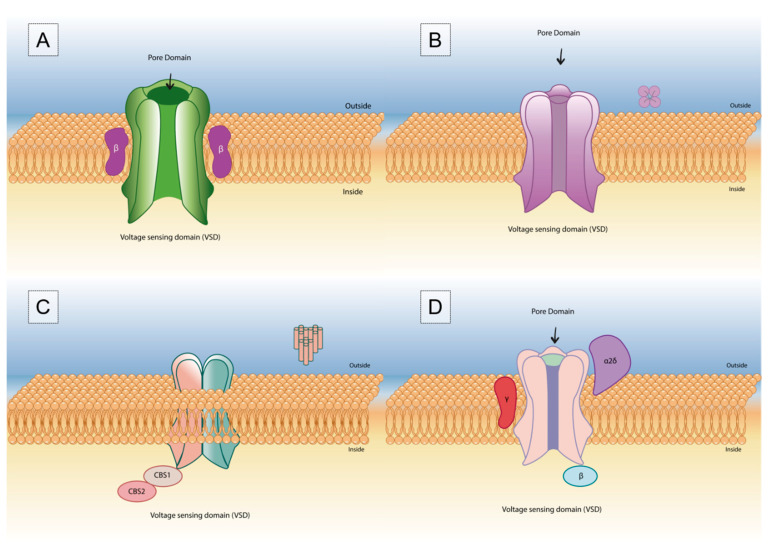
Representing the construction of voltage-gated channels: (**A**) sodium, (**B**) potassium, (**C**) chloride, and (**D**) calcium.

Ion channels (Figure 1) are present in animals, plants, and bacteria, and they are responsible for the regulation of ion flow across cell membranes [44]. They are macromolecules that, under stimuli, will produce a response that allows the selective flow of ions across cell membranes [44,45]. With a fundamental role in physiological processes, ion channels are responsible for controlling chemoelectric signals that coordinate heartbeat, cell cycle, and circadian rhythm, and they modulate the immune response and support neuronal communication pathways [46]. These channels play a role in membrane potential generation, the release of neurotransmitters, signal transduction, muscle contraction, hormone secretion, the sensation of physical and chemical stimuli, motility, and cell growth [10,46].

They can be classified according to their ion selectivity, homologous sequence, and the gating mechanism for both opening and closing. Gating channels can be divided into three groups: voltage-gated channels, ligand-dependent channels, and mechanically sensitive channels [47].

In mammals, voltage-gated Na^+^, K^+,^ and Ca^2+^ channels have similar structures and are usually constituted by a pore-forming subunit or α subunit, but the main difference is that the α subunit in Na^+^ and Ca^2+^ channels is formed by four linked domains (DI-IV), while in K^+^ channels it is formed by tetramerization of four individual domains [48,49].

Each domain of the α subunit consists of six transmembrane (TM) α-helical segments (S1–S6) [50], which in turn are divided into a voltage-sensing domain (VSD), which consists of S1–S4 segments, with S4 containing positively charged residues [48,49], and a pore-forming domain (PD), which comprises S5 and S6 segments separated by a long reentrant loop [48,49,51,52,53,54]. 

The four PDs clustered together in the pore are surrounded by four VSDs. When in the closed or deactivated non-conductive state, the entrance of ions is prevented by the intracellular activation gate, located at the intersection of the four S6 helices, with the opening and closing of the gate being controlled by the VSD [48,49]. The outward or inward movement of the S4 segment following changes in membrane potential induces conformational changes that result in the opening or closing, respectively, of the channel pore [51].

Selectivity for specific ions is enabled by the selectivity filter, which is composed of conserved residues specific for each channel and situated at the pore-lining loops (P-loops) connecting S5 to S6 in the domains [48]. In sodium channels, four amino acid residues (DEKA), present in an equivalent position in each of the domains, control selectivity for sodium [55], and in potassium channels, the selectivity filter is composed of the conserved signature sequence TVGYG in the P-loop [56,57].

In voltage-gated Na^+^ channels (Nav), fast inactivation is mediated by the short intracellular loop connecting homologous domains III and IV of α subunit by folding into the intracellular mouth of the pore and blocking it [55], while in voltage-gated K^+^ channels (Kv), this particle is located at the N-terminus of each subunit and is therefore called N-type inactivation [58,59]. In the voltage-activated Ca^2+^ channels (Cav), occlusion of the pore occurs by the binding of the linker between domains I and II to the cytoplasmic termination of the S6 segment of domains II and III [60].

Many voltage-gated ion channels (VGICs) include, in addition to the α subunit, one or more auxiliary subunits that modify their expression levels, folding efficiency, functional properties, or subcellular localization [61]. Nav channels have two auxiliary subunits, β1 and β2 [61]; Cav channels have at least four β subunits (Cav β1–4), one of at least four α2δ subunits (Cav α2δ1–4), and in some cases, one of several putative γ subunits (Cav γ1–8) [60]; and Kvs have an auxiliary β peptide [62].

Within the Nav and Kv channel families, most of the binding sites for drugs or toxins are surrounded by water molecules and located inside or outside the channel pore, but some compounds can bind to one or more sites that do not fit into these hydrophilic binding sites, creating a pore blockage [49].

Sodium channels are integral membrane proteins that function as a gate for the selective permeation of sodium ions through biological membranes, where they are responsible for the rapid influx of sodium that starts the activation phase of the action potential in nerves, muscles, and endothelial cells [25]. 

The sodium channels subtypes Nav 1.1, Nav 1.2, Nav 1.3, and Nav 1.6 are present in the central nervous system, so the genes SCN1A, SCN2A, SCN3A, and SCN8A, responsible for encoding these sodium channels, respectively, demonstrate their link to the early onset of neurodegenerative diseases and epilepsy [10,63]. On the other hand, the subtypes Nav 1.7, Nav 1.8, and Nav 1.9 are mainly in the peripheral nervous system, while the subtype Nav 1.4 is found in skeletal muscle and Nav 1.5 in the heart [9]. 

Mutations in these channels, as in their pore-forming α subunit and auxiliary β subunits, are associated with human diseases, and because of their physiological role, the channels are promising targets for drugs for the characterization of channelopathies related to pain, epilepsy, and cardiac syndromes [10,64,65]. For example, the cardiac sodium channel Nav 1.5 may have genetic defects related to rare variants present in one or more genes related to the channel, leading to loss or gain of channel current function, which leads to the appearance of disease phenotypes, including Down syndrome, Brugada syndrome, long QT syndrome, and progressive cardiac conduction disease [49]. Moreover, mutations in arginine residues in the S4 segment are associated with different phenotypes in turn correlating with channelopathies of this channel [66].

The family of potassium channels is larger than the Nav channel family, due to the high diversity of genes that can encode different subunits that mix and match to form functional potassium channels [48]. They participate in cell proliferation and cell cycle transition, including being able to influence cancer progression and cell apoptosis [67].

In humans, they are subdivided into five structural and functional groups [68]. The channels known as inward rectifiers (Kir) have two TM segments with a pore-forming loop (P) between them. They can be homo- or heterotetrameric complexes, and their function is regulated by phosphorylation, phosphatidylinositol 4,5-bisphosphate, G proteins, and nucleotides [69]. Two-pore potassium channels (K2P) are regulated by factors such as pH, temperature, and cell membrane tension, and they have four TM segments [70]. Kvs are formed by four identical or homologous α subunits 72. Kv1.3 channels are known to be the target of peptides, mainly those present in scorpion venoms, and have been demonstrated in animal models their relationship to autoimmune diseases such as rheumatoid arthritis, psoriasis, and multiple sclerosis, as well as to inflammatory diseases such as Alzheimer’s and Parkinson’s disease [31]. Calcium-activated intermediate (IKCa) and small-conductance (SKCa) potassium channels are activated by Ca^2+^ via a calmodulin-mediated mechanism, and the S4 segment is almost insensitive to potential changes [71]. They are formed by 6 TM segments (S1–S6) and the pore region (P) located between S5 and S6. High-conductance potassium channels (BKCa) are formed by two members with seven TM segments and are activated by changes in potential and also by ions (Ca^2+^, Na^+^, or Cl^−^) [72].

Ca^2+^ plays a fundamental role in the contraction of cardiac and skeletal muscles, being released from the sarcoplasmic reticulum into the cytoplasm through ryanodine receptors (RyRs) [73], which are present in various types of cells, such as neurons; exocrine cells; smooth, cardiac, and skeletal muscle cells; epithelial cells; endocrine cells; and lymphocytes [35].

Calcium channels can be divided into six transient (T-), long-lasting (L-), N- (neural), Purkinje (P-), (Q-), and residual (R-) classes [74]. They are composed of the pore-forming α1 subunit, the binding sites for drugs, and auxiliary subunits such as β, α2δ, and γ, responsible for the anchoring, traffic, and regulatory functions of the channel [74]. In the literature reviewed, there is no apparent explanation for the name of the calcium channel (i.e., Q-), unlike for other channels, where it is hypothesized that it is just an alphabet convention.

However, T-type Ca^2+^ channels are responsible for the regulation of neuronal excitability, and the increase of intracellular Ca^2+^ causes a depolarization of the membrane, leading to action potentials. They are overexpressed in different types of cancers, which makes them a target for the development of new therapies, where the identification of channel expression, induction of apoptosis, and inhibition of cancer cell proliferation are some of the possibilities to be explored for this type of channel [75].

L-type calcium channels can be subdivided into three subfamilies, and at synapses, they have the following functions: Cav 1 is located in the postsynaptic dendrites and dendritic spines, with calcium input causing long-term potentiation; Cav 2 is present in the presynaptic nerve terminal and has the function of calcium input, leading to neurotransmitter release; and Cav 3′s mutations can alter its function and or its regulation, causing neuropsychiatric diseases such as autism, schizophrenia, bipolar disorder, and depression [76]. Mutations in the CACNA1C gene, due to increased Cav 1.2 activity, cause severe cardiac arrhythmias and neurological and neuropsychiatric abnormalities that are called Timothy syndrome [77]. 

Neurotransmission begins with presynaptic Ca^2+^ channels transmitting P/Q-, N-, and R-type currents, and in the nervous system, P/Q- and N-type channels are pathways for Ca^2+^ entry, which initiates the rapid release of classic neurotransmitters (glutamate, acetylcholine, and GABA). This interaction requires protein engineering with intracellular domains responsible for signal transduction [78]. Therewith, P-/Q-type Ca^2+^ channels are located closer to the axon terminal and to the release zone, leading to higher presynaptic and often clustered Ca^2+^ concentrations in vesicles [79]. The N-type channel is responsible for the release of neurotransmitters from cortical and hippocampal synapses [79]. On the other hand, R-type channels are only partially characterized and are encoded by the CACNA1E gene; it has been observed that several mutations occurring in this gene are clustered in the cytoplasmic ends of the four S6 transmembrane segments, constituting the activation gate of this channel [80].

The diversity of subtypes of calcium channels is mainly to exert specific physiological functions; the variants in the same subtype are a mechanism for their different biophysical properties. Therefore, blockers of this channel are effective for the treatment of anxiety, drug dependence, pain, Parkinson’s disease, and epilepsy [77].

Chloride channels are homodimeric structures with anion-binding sites in the center of each subunit [81]. They conduct Cl^−^ anions and other ions, such as nitrate, thiocyanate, and bicarbonate, and play roles in cellular and physiological activities, such as cell growth, pH regulation, and the transport of solutes of organic nutrients. They can also be found in intracellular organelles, including the mitochondria of cardiomyocytes and exosomes [82,83].

Some chloride channel (CLC) proteins passively conduct Cl^−^, while others are secondary active transporters that exchange two Cl ions for one H^+^ [84]. It is a dimer protein, and the structure of the subunits is formed by 18 α-helices (A-R), 17 of which are partially embedded in the membrane, and each subunit has a triangular shape that interacts with a hydrophobic interface [81].

The transmembrane block has an intracellular carboxyl terminus that contains two cystathionine-β-synthase (CBS) domains, which bind with the intracellular face of the transmembrane block of the same subunit and with the neighboring monomer. The A-I helices have the membrane orientation reversed from that of the J-R helices [81,85]. The roles of CBS1 or CBS2 as well as the binding regions are difficult to identify because mutagenesis at these ends leads to structural changes [81].

Among the mammalian plasma membrane Cl^−^ channels are ClC-1, ClC-2, and the two isoforms of the ClC-K channel [81]. ClC-1 is specific to skeletal muscle, being decisive for tissue excitability, while ClC-K is present in epithelial cells of the nephron, salivary glands, and the inner ear, with a function in transepithelial transport [81]. ClC-2, on the other hand, is expressed in epithelial and non-epithelial cells, has diverse and less defined functions, and may exert the function of regulating cell excitability [81].

However, ClC-3 to ClC-7 channels are considered vesicular 2Cl^−^/H^+^ exchangers and are believed to assist in the acidification of intracellular vesicles [81]. ClC-3 is mainly expressed in the endosome, and it can alter the vesicular voltage or lead to luminal chloride accumulation; it affects vesicular functions, such as vesicle fusion and traffic, and secondary active ion transport [81,86].

ClC-4 plays a slow-emerging role, being homologous to ClC-3 and ClC-5, and it can be found in many tissues, being eminent in the brain with higher expression in pyramidal cells and the hippocampus. The function of CIC-4 is thought to be related to ion homeostasis [81,87]. ClC-5 is kidney-specific with activity in endosomal membrane transport, playing an essential role in receptor-mediated and fluid-phase endocytosis [81].

Accordingly, ClC-6 is considered a late endosomal glycoprotein and is expressed in the brain; in adult mice, it has been detected in the hippocampus in the cortical layers [88]. Meanwhile, ClC-7 is a lysosomal antiporter that is mutated in osteopetrosis and neurodegeneration. It has the general structure of ClC channels; only the stretch between the CBS1 and CBS2 domains is shorter compared to the other channels. Its subcellular localization in mice was found in fibroblasts, neurons of the hippocampus, and proximal tubule cells [89]. Dysfunction in this channel leads to lysosomal storage dysfunction and neurodegeneration [90].

Additionally, the intracellular chloride proteins are classified into six members [91]. They are soluble globular proteins and integral membrane proteins with ion channel activity. They are found in organelles such as mitochondria and can modulate mitochondrial physiology through the generation of reactive oxygen species (ROS) and the capacity of calcium [92]. 

Transient receptor potential (TRP) channels consist of identical or homologous tetramers [93]. Each of the four subunits in the tetramer comprises six TM domains with intracellular carboxy and amino termini [94]. Belonging to this superfamily is the transient receptor potential vanilloid 1 (TRPV1) [95]. Non-selective thermosensitive cation channels that are associated with pain and inflammation are central ligands that are found in both the spinal and supraspinal regions, as well as in peripheral nociceptors. The sensitization of these channels can be induced by painful stimuli, and both central and peripheral sensitizations can lead to the development of inflammatory diseases [96]. 

TRPA1, known as the wasabi receptor channel, is one of the main sensors for irritating and harmful substances, such as the allyl isothiocyanate component present in mustard and onions. It is permeable to Ca^2+^, which leads to neuronal depolarization and channel activation, causing pain and hypersensitivity [42].

The structure of the homotetrameric TRPA1 channel is composed of the transmembrane domain (TMD), which is composed of the voltage sensor-like domain (VSLD; S1–S4), the pore domain (S5, S6, and pore helices), and the terminals (N- and C-) form the two-layer cytoplasmic assembly. The coupling domain (CD) is composed of eight short helices (H1–H7 and pre-S1), a β sheet (βCD) composed of three β strands (β1.1–β1.3), the TRP-like helix, and the ankyrin repeat domain (ARD) (AR12–AR16) with four subunits surrounding the central tetrameric C-terminal coiled-coil [97].

## 3. Neurotoxins

Scorpion neurotoxins interact with Na^+^, K^+^, Ca^2+^, or Cl^−^ voltage-gated ion channels (VGIC) of excitable cells [98]. They are peptides that have conserved folds due to the presence of cysteine bridges but with different functions due to their diverse conformation and charges, resulting in channel-specific receptors with a high binding affinity [7]. Their specificity is so great that peptides with 96.72% similarity in their residues (or two amino acid substitutions) show a major disparity in interaction, making them lethal and/or responsive at the nanogram level [99].

Neurotoxins act either on the voltage-dependent gating or on the pore—blocking it [100]—causing channel inhibition. Moreover, such toxins are able to modify the gate, acting during its opening or closing, depending on the peptide [98,101].

### 3.1. Na^+^ Channel Toxins

**Figure 2:**
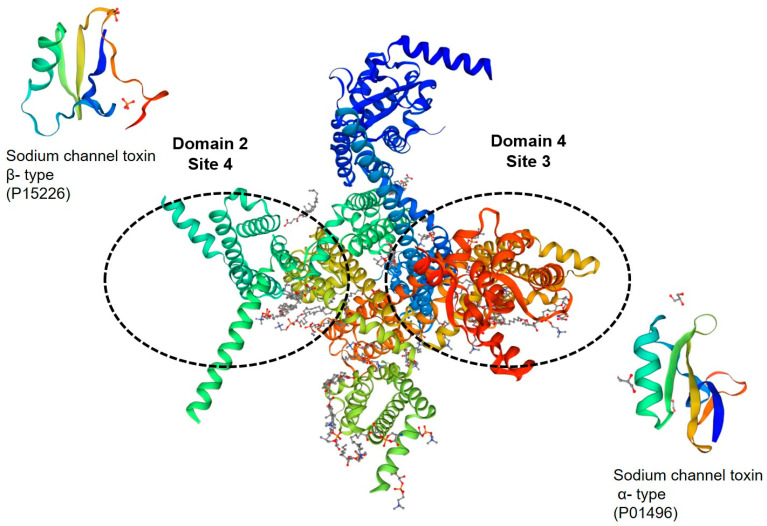
A 3D image created by SWISSMODEL of the Nav channel, in the center, with respective binding sites identified for the following examples of peptides α–type (P01496) and β–Type (P15226), lateral to their binding sites.

Toxins with actions on sodium channels stand out for having effects on mammals, including humans [73]. They are divided into two main types (Figure 2): α-toxins are responsible for slowing down the fast inactivation of the channel, inhibiting the inactivation phase of the action potential, and β-toxins cause a shift in the membrane potential dependence of the activation channel to more negative potentials, which ends up reducing the amplitude of the peak current [102,103,104]. Both α-toxins and β-toxins lead to the greater entry of sodium into cells [103]. Activation of these channels and depolarization cause a massive release of neurotransmitters, mainly glutamate [105], but specific toxins have also been shown to act specifically on the dopaminergic system [106].

#### 3.1.1. α-Toxins

α-Toxins are generally more abundant in the venom of the family Buthidae. Due to their neurotoxic effects, α-toxins are also known as “potential dependent”. They are present in paleotropical scorpion venom and act only when the channel is open. Their binding affinity is proportional to the polarization intensity of the membrane, and they inactivate the closure of the sodium channel without modifying its opening potential [104,107].

They are peptides containing 60 to 70 amino acid residues with four disulfide bonds [108,109], which bind to sodium channel receptor site 3, located in the extracellular segment S3–S4 of domain IV of the channel [100,110].

The venoms of *Androctonus* have high-affinity ligands for sodium channels responsible for cellular excitability, with α toxins being the main peptide responsible for their lethal effect. Their action occurs in the type IV domain, present in neuronal and muscular sodium channel subtypes of mammals, which will act as a sensor responsible for the opening movement, delaying its inactivation, and causing membrane depolarization [25,111].

The α-toxins can also be divided according to their activity in sodium channels of mammals and insects; thus, they can be called “classic” as they act only on ion channels of mammals and “α-like” insect toxins that have their action on both insect and mammal channels. Examples of classic toxins are Lqh2 from the scorpion *Leiurus quinquestriatus hebraeus*, which is highly toxic in mammalian brains [23]; and LqhaITs and Lqh3 are examples of α-like toxins (*Leiurus quinquestriatus hebraeus*) [24,25].

The genus *Centruroides* includes dangerous species distributed throughout the Americas, with a great incidence in Mexico and Colombia, including parts of the western USA and the northern United Mexican States [112,113,114]. The CvIV4 peptide was isolated from the venom of *Centruroides vittatus* scorpions, and it acts mainly on Nav 1.2, Nav 1.3, Nav 1.4, and Nav 1.7 channels, causing acute pain [115].

#### 3.1.2. β-Toxins

β-Toxins bind to site 4 of the channels, shifting voltage-dependent activation to more hyperpolarizing potentials and reducing the peak amplitude of the sodium current [110,116]. They are composed of 60–65 amino acids and four disulfide bonds [117].

Acting on domain II in the extracellular loop of the S3–S4 segments [118], β-toxins have a bimodal function, as they can increase the excitatory mode and inhibit the depressive mode of Nav channel activities [119].

During its activation, the voltage sensors are externally translocated, allowing the interaction of the toxin with the extracellular portion of the segment. They are responsible for inducing spontaneous and repetitive triggering of the action potential, which allows Nav channels to be activated at subthreshold membrane potential; however, they also reduce the peak current of the Nav channel [78,102].

Ts1 is the most toxic major peptide present in the venom of the scorpion *Tityus serrulatus*. It is a classic β-toxin that acts at site 4 of the sodium channel [120] and modulates the Nav 1.3 to Nav 1.6 sodium channels [29]. However, electrophysiological studies have shown that it also acts as an α-toxin, being toxic to mammals and insects [28,118]. Ts1, also known as the γ-toxin present in this scorpion’s venom, blocks the opening of the channel as well as its closure when it is open, thus exerting a combined effect [121]. This toxin can also cause the activation of the cascade of events that lead to the secretion of mediators (cytokines and nitric oxide) because of the binding of the toxin to the receptor, leading to secondary actions that can lead to the death of the victim [26].

### 3.2. K^+^ Channel Toxins

Among peptides that affect the K^+^ channel, there is Vm24 (α-KTx 23.1), acting on the Kv1.3 channel, which is a peptide derived from HsTX1 and its analog HsTX1[R14A] from the scorpion *Vaejovis mexicanus smithi* and responsible for blocking this channel. Kv1.3 channels are overexpressed in effector memory T cells and are involved in the prevention of neurological symptoms that can appear in multiple sclerosis when the destruction of the myelin sheath of the nerves occurs. Thus, potassium efflux blockers are important for the prevention of neurological symptoms [21,30,31].

The scorpion *Buthus martensii Karsch* has a peptide, BmK86-P1; it has six cysteine residues in its structure, acts on the Kv1.2 channel, and may be a target for new drugs for ataxia diseases, which affect the coordination of movements, and for epilepsy [32]. 

**Figure 3:**
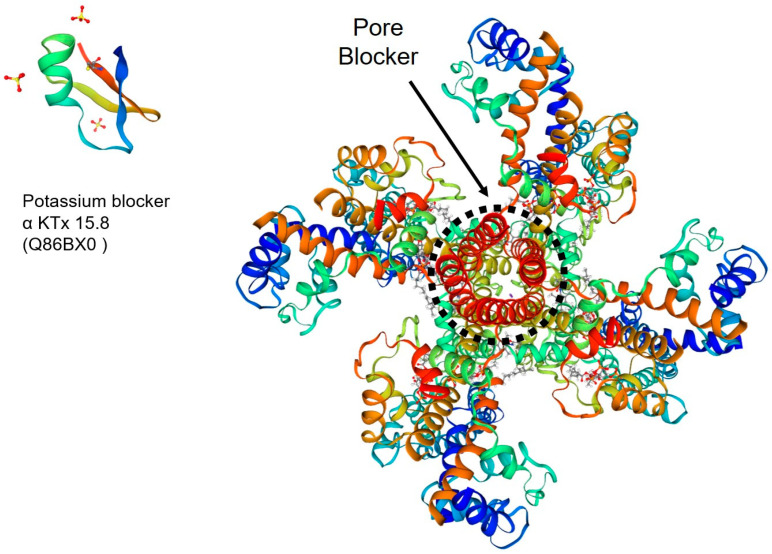
A 3D image created by SWISSMODEL of the Kv channel, with the respective pore blocker peptide identified as α KTx 15.8 (Q86BX0), as an example.

Short-chain peptides with 30–40 amino acid residues are specific blockers of Kv channels (Figure 3), as in the case of charybdotoxin (CTX) isolated from the venom of the scorpion *Leiurus quinquestriatus*. It acts by binding the external part of the pore that prevents the conduction of K^+^ ions without a conformational deformation [33,122,123].

The Kv11.1 channel, known as human ether-a-go-go (hERG1), is one of the most studied and plays an important role in the heart, brain regions, endocrine cells, smooth muscle cells, and tumor cells [67,124]. CMERG1 is a peptide isolated from the Colombian scorpion *Centruroides margaritatus* that is responsible for the total blockade of the current of potassium channels in the hERG1, which is responsible for the repolarization of the cardiac action potential; alterations in this channel are associated with cardiac disorders; this peptide is considered more stable than the toxin ɣ-KTx1.1, isolated from the scorpion *Centruroides noxius* [34].

### 3.3. Ca^2+^ Channel Toxins

Scorpion venoms also affect the release of Ca^2+^ stored in the sarcoplasmic reticulum of skeletal and cardiac muscles. They act by binding to ryanodine receptors, which are calcium channels present in endoplasmic and sarcoplasmic reticulum membranes, regulating the intracellular free calcium concentration. Intracellular calcium triggers events that lead to muscle contraction, hormone secretion, lymphocyte activation, fertilization, and other physiological processes [125]. Calcium channels act as calcium influx mediators through plasma membrane depolarization or Ca^2+^ binding channels, which control its release from intracellular stores [126].

Calcins act as agonists of ryanodine receptors (RyRs), which are calcium channels present in the endoplasmic reticulum and sarcoplasmic membranes [127]. Isolated from *Tityus serrulatus* venom, the Ts peptide shows cellular penetration capacity, increasing the release of intracellular Ca^2+^ by the activation of the nuclear InsP3R receptor present in cardiomyocytes [128]. We should highlight important features of this peptide that make it attractive from a biotechnological point of view. Its amino acid sequence is different from other calcins, having basic residues terminated by Ser or Thr, whereas CPP-Ts have negatively charged neutral or apolar amino acids and are able to be internalized by cancer cells but not by normal cell lines [128].

For the interaction between calcins and RyRs, the hypothesis is that the positively charged basic residues stay on one side of the calcins, forming a dipole, which will interact with the negatively charged lipids of the membrane; this interaction occurs with the hydrophobic regions of the toxin and the inner membrane, favoring the passage of the toxin [127].

Imperatoxin A (IpTxa) is a peptide isolated from the venom of the African scorpion *Pandinus imperator* that is capable of reversibly activating this channel [35]. It appears to be the first identified toxin that activates the calcium channel [8]. It can cross cell membranes and alter the release of Ca^2+^ and transport an impermeable charge across the membrane, where this could be an option for drug delivery [35]. It is a peptide modulator with a high affinity for RyR1 (skeletal), increasing its opening time and the binding of RyR1 [129]. 

Similar scorpion venom peptides have been identified: maurocalcin (MCa) from *Scorpio maurus palmatus*, hemicalcin (HCa) from *Hemiscorpius lepturus,* and hadrucalcin (HdCa) from *Hadrurus gertschi*; these peptides have 33–35 amino acids with 6 cysteine residues [36,37,38]. They are responsible for the interaction with and stimulation of RyR1, leading to a substantial release of Ca^2+^ in the sarcoplasmic reticulum [8,36].

Kurtoxin, a toxin isolated from the African scorpion *Parabuthus granulatus*, potently inhibits the voltage-dependent T-type calcium channel in mouse male germ cells, leading to the impediment of the acrosome reaction and preventing fertilization in the egg [39]. The peptide AaTs-1, obtained from the venom of the scorpion *Androctonus australis*, demonstrated antiproliferative action against human glioblastoma cells, being about twice as active as the drug temozolomide. It was found to affect the MAPK pathway, which increases p53 expression and leads to the alteration of the cytosolic concentration of calcium; calcium acts as a second messenger, and its intracellular increase influences the proliferation and migration of glioblastoma cells by modulating the formyl peptide receptor-like 1 (FPRL-1) [41,130].

Currently, three main classes of Ca^2+^ channel drugs are used, namely dihydropyridines, phenylalkylamines, and benzothiazepines, which bind in the region overlapping the pore and the activation gate [74]. Uncharged dihydropyridines act mainly on cardiac Cav 1.2, inducing or stabilizing the inactivated channel state [131]. In contrast, phenylalkylamines and benzothiazepines bind to open and inactive states, stabilizing the inactivated channel states and delaying inactivation [131].

As we can see, there are several drugs in use with a target in the calcium channel, but the discovery of compounds that have high affinity and selectivity and can cross the blood-brain barrier are not rapidly metabolized; pursuing the maintenance of physicochemical properties that are both adequate and non-toxic can be an extremely challenging endeavor [77]. From the point of view of the development of new drugs, functional diversity such as alternative splicing specific to the genes of these channels and the use of approaches such as the association of targeting protein interaction must be considered [77].

### 3.4. Cl^−^ Channel Toxins

Cl^−^ channels are essential in cellular physiology, acting in the stabilization of cell membrane potential, transepithelial transport, maintenance of intracellular pH, cell proliferation, fluid secretion, and regulation of cell volume [82]. Given its importance in cellular functions, disorders caused mainly by mutations in its gene may trigger pathologies such as neurodegeneration, leukodystrophy, mental retardation, deafness, blindness, myotonia, hyperaldosteronism, renal salt loss, proteinuria, kidney stones, male infertility, and osteopetrosis [81]. There are gaps in the characterization of these channels, which makes it difficult for them to be presented as targets for pathological conditions [82].

The main toxin characterized as affecting this channel is chlorotoxin (ClTx), obtained from the venom of the scorpion *Leiurus quinquestriatus*. It is a peptide considered a short-chain peptide formed by 36 amino acids with disulfide bonds and 8 cysteines [40,132]. Frequently utilized in research to investigate the correlation between the toxin and the chlorine domain and to comprehend the mode of action of this toxin on the vascular endothelial growth factor receptor neuropilin-1 (NRP1) domain [132].

As a potential anticancer agent, chlorotoxin inhibits the influx of chloride into glioma cells. It also binds to matrix metalloproteinase 2 (MMP-2), which is overexpressed in gliomas [133]. The ClTx peptide has been used to increase the effectiveness of current antitumor drugs and combined with other drugs, nanoparticles, radioisotopes, and fluorescent molecules. The compound 131 I-TM601 even reached a phase III clinical trial, identifying glioma cells through radiation [134].

Despite the proven role of chloride in apoptosis and cell proliferation, there is a lack of understanding of the complex cellular and molecular signaling involved in disease processes such as cancer, as well as a lack of information regarding the identity of this channel, thus hindering the development of new therapeutic targets [82].

The gap can be filled with future studies as well as the use of techniques to understand the structure and function of this channel, identifying possible therapeutic targets. The description, mainly of chlorotoxin, proves to be a problem faced in general for the bioprospecting of new drugs since the venom of this specific scorpion does not have a description of other peptides, which could be the key or the way to discoveries until then.

### 3.5. TRP Channel Toxins

The peptide WaTx from the Australian black rock scorpion *Urodacus manicatus* acts on the prolonged opening of the TRPA channel, consequently leading to neuronal depolarization and hypersensitivity, causing pain and hypersensitivity but not neurogenic inflammation [42].

Hakim et al. [43] demonstrated that the peptide BmP01 from the scorpion *Mesobuthus martensii* induces pain similarly to the agonist drug of this channel, capsaicin. They obtained significant results by showing that the toxin has its effect diminished under acidic conditions when acting on the Kv1.3 channel, but in the TRPV1 channel, its effect is potentiated.

Among the obstacles found in the studies of this channel and the development of possible antagonists to block the transmission of pain, there is the blockade of several nociceptors, not just specific molecules, and the side effects of the molecules, leading to sensations such as hyperthermia [135,136]. It has not yet been elucidated how the modulation of the BmP01 peptide and its passage in the channel actually occur, or whether the Kv1.3 channel also contributes to its activation. There was also no test for the specificities such as sensitivity to heat and tactile stimuli or desensitization after the action of the peptide [137].

The receptor properties of TRPV1, being polymodal and activated by heat, lipids, and voltage, make it an attractive target for the development of pain inhibitors, but it has serious side effects, such as hyperthermia, which affects most patients. Tests with peptides end up failing in both animal and human clinical trials [138]. It is worth mentioning that it is possible to develop modality-dependent TRPV1 inhibitors related to pain with the use of specific peptides, mainly peptides that are rich in disulfide bridges and that have not yet been explored [138].

## 4. Peptides and Other Applications in Ion Channels

The development of analgesics that act mainly on voltage-gated sodium channels has been studied because they are an attractive target for the development of new drugs and they have a direct link with the generation and propagation of pain [139]. In this aspect, scorpion peptides have proved to be an interesting target, as they present fundamental studies for the identification and characterization of ion channel subtypes that can result in both analgesia and pain, mainly sodium ion channels such as Nav 1.7, Nav 1.1, Nav 1.3, Nav 1.6, Nav 1.8, and Nav 1.9 [139].

The peptide BmK AGAP, isolated from the venom of the scorpion *Buthus martensii Karsch*, has analgesic and antitumor activity and exhibits a role in cell cycle arrest and apoptosis. This peptide is an ion channel regulator, acting on calcium channels activated by high and low voltage and on sodium channels resistant to tetrodotoxin [27]. Some challenges still have to be overcome for the application of toxins present in *Buthus martensii Karsch* (BmK) venom for the treatment of neurological disorders; among them are their applicability and characteristics of the molecule itself, such as hydrophilicity, size, polarity, and ability to penetrate the blood-brain barrier [140]. However, for the peptide IMe-AGAP of the Iranian scorpion *Mesobuthus eupeus,* responsible for the inhibition of sodium channels Nav 1.8 and Nav 1.9, Dehghan and collaborators demonstrated great structural similarity. Through docking analysis, they observed about (93/85%) similarity with the peptide BmK AGAP, maybe making it a target for future work in the development of pain treatment [141]. These results show that despite the structural similarity, the molecular difference between these peptides may lead to different behavior, which may be important for their application, including their pharmacological action.

Another application of Kurtoxin would be as an antidepressant through viral expression. It can suppress N-methyl-D-aspartate (NMDA) receptors; in view that depression symptoms are caused by dysregulation of neuronal activities and synaptic transmission, the use of a viral vector with the expression of a peptide can modulate T-type Ca^2+^ ion channels, compensating for its dysregulation caused by synaptic depression in the lateral habenula [142]. It can also have an antiepileptic effect through its expression by a viral vector, in which case it is expected that when injected, it will show this effect because the cells will secrete peptides that will be responsible for blocking the channel and suppressing excessive neuronal activity [142].

One of the biotechnological applications of scorpion venom is its use as a bioinsecticide [73]. The selective insect toxin of the scorpion *Androctonus australis Hector* (AaIT) could be used as a pest biocontrol tool. Formed by 70 amino acids and 4 disulfide bridges, it specifically targets the sodium channels of insects, leading to rapid paralysis and death due to its binding to the motor nerve terminal, which causes excitation and stimulation of skeletal muscles and leads to rapid paralysis [27,143,144].

In this perspective, peptides from arachnids and spiders are also important, as are studies in the area of bioprospecting for new products as commercial products are being developed. The insecticide from the venom of the Australian funnel web spider, the neurotoxin GS-ω/κ-HXTX-Hv1a, has its action on BKCa and Ca^2+^ channels. It could be interesting for pest control in agriculture since it targets different insect orders, such as Coleoptera, Lepidoptera, Diptera, Orthoptera, Thysanoptera, and Hemiptera [145,146]. Among the toxins found in spider venoms that target insects are μ-NPTX-Nc1a from the *Nephila clavata* spider, which targets the American cockroach *Periplaneta americana* [147], and Magi3 from *Macrothele gigas,* with action on *Gryllus bimaculatus* [148].

Some scorpion toxins may have specific antiviral effects against several different viruses, such as mucroporin-M1, a mucroporin derivative from *Mucronatus lychasvenen*, with antiviral activity against three RNA viruses, measles (MeV), coronavirus (SARS-CoV) and influenza H5N1 [127].

The studies carried out by Fakih [149] sought to find therapeutic agents that can prevent the spread of COVID-19 infection through a computational approach with the use of antimicrobial peptides present in the venom of the scorpion *Lychas mucronatus*, testing mucroporins for receptors specific for SARS-CoV-2, which is the major protease (Mpro).

The analysis of structural and molecular data has been adding and highlighting important steps for the future, aligned with new technologies, which can be of great use for science, but there is still a long way to go when it comes to in vivo testing. We realize that there is still a great demand for isolated peptides to reach clinical testing and then clinical use.

## 5. Conclusions

Conceptually, the exchange of ions constitutes the most basic physiological balance of the cell, and at some level—even mitochondrial—all cells have ion channels that exchange with the environment; the possibility of regulating the channels that permeate them leads to the essential control needed to remedy various disorders, channelopathies, and degenerative diseases. In this ocean of knowledge about neurotoxins, we present just a drop of this vast universe. Here, we compile the main features discussed so far on the subject, joining the basic concept of channel functioning to the most diverse application possibilities of channel-modulating scorpion venom peptides. Therefore, despite all the current literature, it is still an area of study that deserves attention for not exercising its full potential. We hope that this review can provide some answers or raise some questions to be addressed in future research.

## Figures and Tables

**Table 1:** Examples of peptides isolated from scorpion venom with actions on different channels and proposed mechanisms of action.

	Animal	Peptide	Effect	Mechanism of Action	Binding Site	References
**Sodium channel**	*Leiurus quinquestriatus hebraeus*	Lqh2	Blocks neuronal transmission	Blocks inactivation of activated channels	Site 3	[23]
		LqhaIT	Delays inactivation	Prevents external voltage sensor movement	Site 3	[24]
		Lqh3	Blocks neuronal transmission	Inhibits the inactivation of activated channels	Site 3	[25]
	*Centruroïdes noxius *	Cn2	Cell cycle arrest	Reduction of peak amplitude	Site 3—Nav 1.6	[26]
	*Buthus martensii karsch*	BmK AGAP	Cell cycle arrest in the G1 phase	Inhibits Nav 1.5 mRNA expression	Site 3 Nav 1.5	[27]
	*Tityus serrulatus*	Ts1	Lowers action potential threshold	Holds voltage sensor in the outermost position	Site 4—Nav 1.3 to Nav 1.6	[28,29]
**Potassium channel**	*Vaejovis mexicanus smithi *	Vm24 (α-KTx 23.1)	Prevents neurological symptoms	Blocks hKv1.3/KCNA3 potassium channels of human T lymphocytes	hKv1.3	[30,31]
	*Buthus martensii Karsch *	BmK86-P1	-	-	Kv1.2	[32]
	*Leiurus quinquestriatus*	charybdotoxin (CTX)	-	Bimolecular inhibition process	Outside of the pore	[33]
	*Centruroides margaritatus *	CMERG1	Repolarization of the cardiac muscle action potential	Blocks Kv11.1 channel	Human ether-à-go-go-Related gene (hERG1)	[34]
**Calcium channel**	*Pandinus imperator*	Imperatoxin A (IpTxa)		Induction of opening or its inhibition	Ryanodine receptor types 1, 2, and 3	[35]
	*Maurus palmatus*	Maurocalcine (MCa)	Release of Ca^2+^ in sarcoplasmic reticulum	Stabilizes ryanodine 1 receptor opening—long-lasting subconductance state	Ryanodine receptor type 1 (RyR1)	[36]
	*Hadrurus gertschi*	Hadrucalcin (HdCa)	Release of Ca^2+^ in sarcoplasmic reticulum	Stabilizes ryanodine 1 receptor opening—long-lasting subconductance state	Ryanodine receptor type 1 (RyR1)	[37]
	*Hemiscorpius lepturus*	Hemicalcin (HCa)	Release of Ca^2+^ in sarcoplasmic reticulum	Stabilizes ryanodine 1 receptor opening—long-lasting subconductance state	Ryanodine receptor type 1 (RyR1)	[38]
	*Parabuthus granulatus*	Kurtoxin	Prevents fertilization in the egg	Impedes the acrosome reaction	Inhibits the voltage-dependent T-type	[39]
**Chloride channel**	*Leiurus quinquestriatus*	chlorotoxin (ClTx)	Inhibits proliferation of glioma cells	Inhibits the influx of chlorine	-	[40]
	*Androctonus australis*	AaTs-1	Increase in P53 expression	Via MAPK—alters cytosolic calcium concentration	-	[41]
**Transient receptor potential vanilloid 1 channel (TRPV1)**	*Urodacus manicatus *	WaTx	Pain and hypersensitivity	Prolongs channel opening—neuronal depolarization	Across the plasma membrane	[42]
	*Mesobuthus martensii *	BmP01	Induces pain	Prolongs channel opening—neuronal depolarization	Across the plasma membrane	[43]

## Data Availability

Not applicable.
